# 

**Published:** 1916-06

**Authors:** 


### Contents

Event and Comment .......... 251
Sterilization of Dental Instruments .......... 253
Preventive Dentistry .......... 274
Commercialism .......... 277
Facts Regarding Succinimide of Mercury .......... 280
Shall We Discontinue Devitalization? .......... 282
Obituary .......... 287
Indents .......... 288
Announcements .......... 299
Bibliography .......... 300

